# Interannual Variability in Greenhouse Gas Emissions Challenges Post‐Restoration Net Sink Predictions in California Delta Wetlands

**DOI:** 10.1111/gcb.70700

**Published:** 2026-01-09

**Authors:** Kuno Kasak, Arman Ahmadi, Iryna Dronova, Ariane Arias‐Ortiz, Tianxin Wang, Alex C. Valach, Daphne Szutu, Joseph Verfaillie, Dennis D. Baldocchi

**Affiliations:** ^1^ Department of Environmental Science Policy and Management University of California Berkeley Berkeley California USA; ^2^ Department of Geography University of Tartu Tartu Estonia; ^3^ Departament de Física Universitat Autònoma de Barcelona Barcelona Spain; ^4^ Bern University of Applied Sciences Bern Switzerland

**Keywords:** carbon dioxide, carbon sequestration, eddy covariance, methane, radiative forcing, switchover time

## Abstract

Globally, wetlands can sequester and store large amounts of soil carbon over the long term due to high primary productivity and slow decomposition. Yet centuries of drainage for agriculture and development have turned many of these carbon sinks into greenhouse gas (GHG) sources. Restoring degraded wetlands, particularly in peat‐rich landscapes, is increasingly promoted as a nature‐based solution for climate change mitigation. However, the trajectory and timing of recovery remain uncertain, especially given the complex interplay among vegetation dynamics, hydrology, and GHG fluxes. In this study, we analyzed 44 site‐years of continuous eddy covariance measurements of carbon dioxide (CO_2_) and methane (CH_4_) fluxes from restored wetlands in California's Sacramento‐San Joaquin Delta. Our findings reveal substantial interannual variability in GHG exchange across sites, driven by differences in restoration design, water management, and vegetation establishment. While rapid vegetation growth, especially dense stands of macrophytes, can enhance CO_2_ uptake, it often elevates CH_4_ emissions and complicates predictions of when wetlands become net GHG sinks. Crucially, wetlands with delayed vegetation establishment due to high or inconsistent water levels (e.g., significant drawdown) remained persistent GHG sources, even years after restoration. Conversely, sites with tailored planting or natural and rapid recolonization exhibited earlier transitions to net sink status, including earlier shifts towards net negative radiative forcing since the restoration. The study highlights the importance of adaptive, site‐specific restoration strategies and long‐term monitoring to capture switchover dynamics from sources to sinks. As global investment in wetland restoration grows, our findings underscore the need to balance climate mitigation goals with ecological realities and the self‐designing processes of vegetation succession.

## Graphical abstract

44 site‐years of eddy covariance measurements over restored California Delta wetlands show strong interannual variability in CO_2_ and CH_4_ fluxes. Vegetation establishment and water management drive whether sites act as greenhouse gas sources or sinks, challenging predictions of post‐restoration climate benefits.

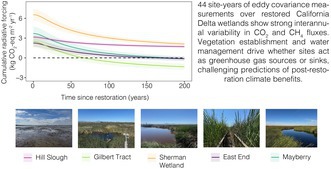

## 1 Introduction

Peatland ecosystems contain almost 30% of the global soil organic carbon stocks, while covering only about 3% of the land surface (Leifeld and Menichetti 2018). However, globally, wetlands have been drained for centuries to accommodate human activities, primarily agriculture, forestry, and urban development (Fluet‐Chouinard et al. 2023). The conversion of these wetlands has produced some of the world's most fertile and productive agricultural lands (Drexler et al. 2009; Reid et al. 2018; Schultz et al. 2005), and therefore the loss of global wetlands over time has been immense (Davidson 2014). This degradation has widespread impacts on terrestrial water storage and flood buffering (Åhlén et al. 2022), evapotranspiration (Sterling et al. 2013), emissions of nitrous oxide (N_2_O) and methane (CH_4_) (Bahram et al. 2022; Saunois et al. 2024), nutrient removal (Kasak et al. 2018), biodiversity (Pasquet et al. 2015), and most importantly, the overall carbon balance (Fluet‐Chouinard et al. 2023). Drainage eliminates nearly all the benefits wetlands naturally provide. Despite decades of reporting on wetland losses, global efforts to restore these degraded ecosystems remain negligible. Many historic wetlands are now so severely altered that full restoration to their original state is extremely challenging or even impossible (Moreno‐Mateos et al. 2012). Nevertheless, rewetting drained soils has been shown to reduce climate impacts overall and is considered one of the most effective nature‐based solutions for long‐term carbon sequestration (Günther et al. 2020; Schuster et al. 2024; Valach, Kasak, Hemes, et al. 2021). The Intergovernmental Panel on Climate Change (IPCC) Sixth Assessment Report emphasized that reversing anthropogenic climate change will require substantial net removals of carbon dioxide (CO_2_) from the atmosphere over a sustained period (IPCC 2023).

In this context, restoring degraded organic peat soils stands out as a particularly promising option for climate mitigation. However, wetlands are among the most challenging ecosystems to restore, and there is no universal solution. Restoration strategies must be tailored to each climate zone, region, and sub‐region as well as traditional and customary rights of land use. What works in boreal climates may not be suitable for tropics or Mediterranean areas, and vice versa. Likewise, tidal and non‐tidal wetland restorations face fundamentally different challenges, from fluctuating water levels to lateral carbon export to adjacent water bodies (Bogard et al. 2020). Therefore, successful restoration depends on the interaction of multiple factors, beginning with the design of restoration plans and extending to the dynamic environmental conditions that influence ecosystem‐scale greenhouse gas (GHG) fluxes. In this case, the primary goal of wetland restoration is to support climate mitigation by converting degraded or disturbed ecosystems, which are often persistent sources of CO_2_, into net carbon sinks (Arias‐Ortiz et al. 2021; Hemes et al. 2019). However, restoration is also expected to deliver co‐benefits such as enhanced biodiversity, water storage, and water purification (Ferreira et al. 2023; Meli et al. 2014). The extent to which these goals are achieved depends heavily on restoration design and allowing time for the self‐designing capacity of nature (Mitsch and Wilson 1996). For example, dense vegetation with macrophytes such as cattails (*Typha* spp.), common reed ( *Phragmites australis* ), and tule (*Schoenoplectus* spp.) can promote high CO_2_ uptake but may also substantially increase CH_4_ emissions, limiting biodiversity (Brix et al. 2001; Hemes et al. 2018; Valach, Kasak, Hemes, et al. 2021) and increasing evapotranspiration (Eichelmann et al. 2018). In contrast, wetlands with a higher degree of complexity (e.g., varying bathymetry) support biodiversity (Elliott et al. 2020; Rannap et al. 2020) but could result in more variable and/or lower net CO_2_ uptake rates. Less vegetated wetlands generally sequester less CO_2_, which can extend the time it takes for a wetland to become a net carbon sink (Schuster et al. 2024; Valach, Kasak, Hemes, et al. 2021).

Climatic conditions and carryover effects of previous land use also play a critical role in both long‐term restoration success and vegetation species selection. In boreal regions, restoration often focuses on *Sphagnum* mosses, *Carex* species, and other species native to these regions (Guo et al. 2016; Kivimäki et al. 2008; Laatikainen et al. 2025; Tong et al. 2025). In the Sacramento‐San Joaquin Delta, for instance, historical wetlands were dominated by species like tule and cattail (Drexler et al. 2009). In warm climates with long growing seasons, these plants can produce large amounts of biomass and contribute to peat accumulation (Hemes et al. 2019; Knox et al. 2015). While emergent macrophytes can take up considerable CO_2_, their high CH_4_ emissions and the prolonged active decomposition period often offset the carbon benefits and delay switchover times when sites become a GHG sink (Hemes et al. 2018). To properly evaluate the ecosystem carbon balance, the eddy covariance (EC) method is one of the most effective solutions, which allows direct measurement of both net ecosystem exchange (NEE) and CH_4_ over ecosystem scales (Baldocchi et al. 1988).

Here, we synthesized 44 site‐years of continuous CO_2_ and CH_4_ flux data from a network of EC towers across different restored wetlands in the Sacramento‐San Joaquin Delta (hereafter the Delta) to investigate the main drivers of interannual variability in CO_2_ and CH_4_ fluxes. The Delta in California, USA represents a suitable study area for the restoration of wetlands following drainage for intensive agriculture. Over the decades, several wetland restoration projects with long‐term EC monitoring provide us with a unique opportunity to investigate how the design of wetland restoration affects vegetation development, carbon uptake, and carbon budgets depending on site‐specific and environmental conditions (Valach, Kasak, Hemes, Szutu, et al. 2021). The continuous NEE measurements show us how much CO_2_ is captured from the atmosphere as gross primary productivity (GPP) via photosynthesis, minus any losses as autotrophic or heterotrophic ecosystem respiration and photo‐oxidation. In addition, continuous CH_4_ monitoring allows us to calculate the net ecosystem carbon balance of each restored wetland and model the timeframe in which the wetlands become net‐GHG sinks. Therefore, the main questions of this study are: (1) what are the main environmental and site‐specific drivers that affect CO_2_ uptake and CH_4_ emissions on annual scales? (2) how do different restoration designs influence the overall GHG budget and the radiative forcing associated with each action? and (3) what would be the optimal post‐restoration monitoring duration to confidently predict switchover times from net GHG sources to sinks.

## 2 Materials and Methods

### 2.1 Study Sites

**FIGURE 1:**
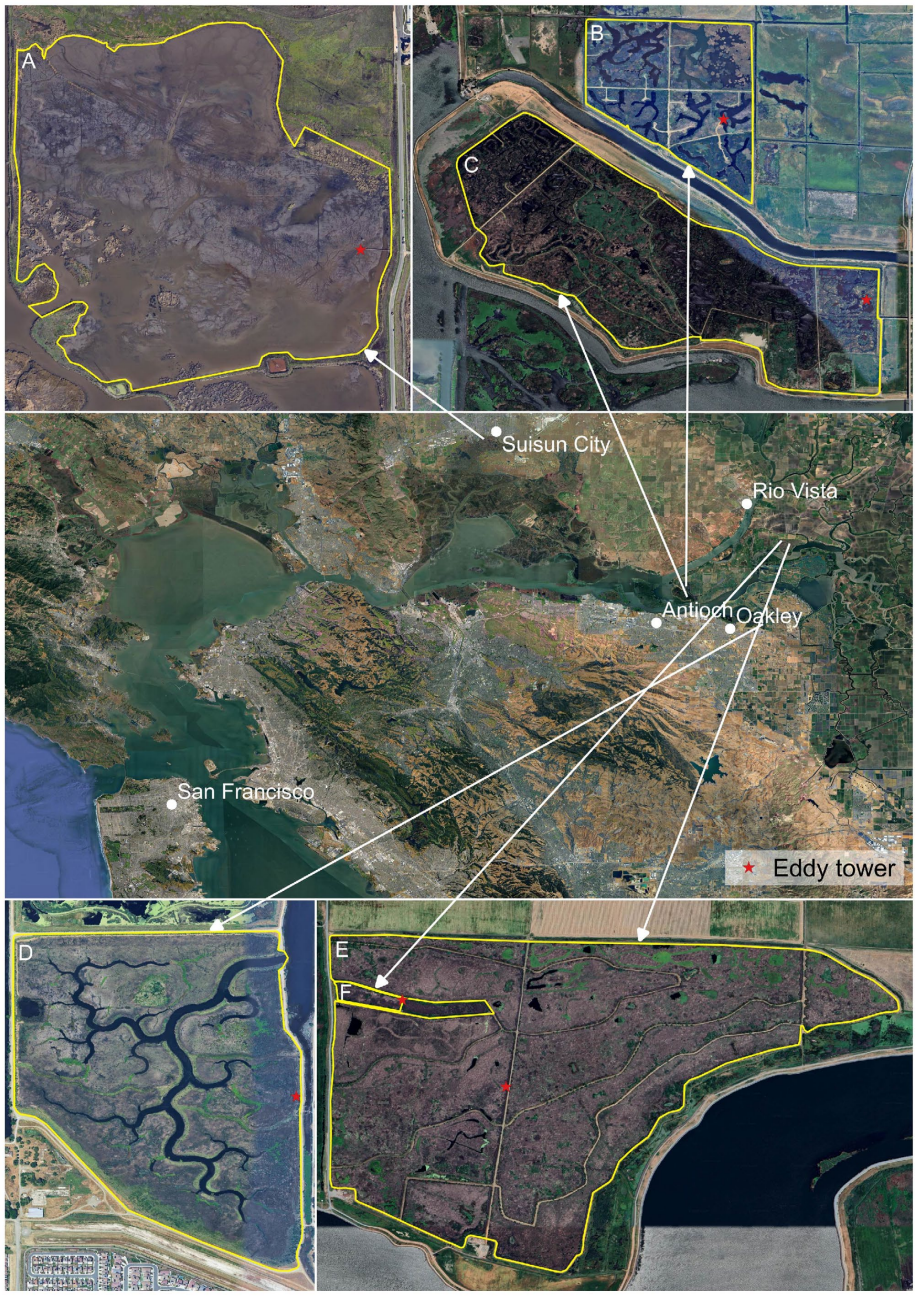
Map outlining the Sacramento‐San Joaquin Delta with the six restored wetland sites: (A) Hill Slough, (B) Mayberry, (C) Sherman Wetland, (D) Gilbert Tract, (E) East End, and (F) West Pond. Eddy tower locations are marked with red star.

The drainage of the vast Delta wetlands started in the 1850s to convert its organic‐rich soils for agricultural use (Cloern and Jassby 2012; Mount and Twiss 2005). Since then, microbial decomposition of peat soils has led to the loss of up to 200 teragrams (Tg) of carbon to the atmosphere (Drexler et al. 2009). Due to ongoing peat compaction and oxidation, some areas of the Delta are now more than 9 m below sea level. This significant land subsidence necessitates constant maintenance of levees and dams to protect the Delta from flooding by the Sacramento and San Joaquin Rivers. However, continued subsidence, combined with hydrostatic pressure from the rivers and sea‐level rise, increases the risk of levee failure—a problem that has occurred multiple times throughout the Delta's history. Restoring subsided lands to wetlands is a promising solution to rebuild soil, reduce subsidence, and stabilize the landscape. Eventually, reconnecting restored wetlands to their natural hydrological regimes is an important first step toward achieving long‐term resilience. Our study sites included both tidal and non‐tidal with fresh and brackish water restored wetlands located in the Delta (Figure 1; Table 1). Tidal sites are influenced by natural tidal cycles and are generally situated on less subsided land. In contrast, non‐tidal sites are more deeply subsided, and their water regimes are actively managed. As a result, water levels are generally aboveground and fluctuate throughout the year, not as a function of precipitation or tides, but due to management interventions.

**TABLE 1:** Summary characteristics of restored wetland sites in the Sacramento‐San Joaquin Delta.

Site (Ameriflux ID)	Previous land use	Year restored/# of years of EC data	Area (ha)	Vegetation establishment	Plant proportion in 2022 (80% flux footprint)	Tidal influence
Gilbert Tract (US‐Dmg)	Pasture	2022/2	165	Planted sections	95.1	Tidal
Hill Slough (US‐Hsm)	Seasonal impounded wetland	2021/3	263	No planting	0.0	Tidal
Sherman Wetland (US‐Sne)	Pasture and pepperweed	2016/3	263	No planting	45.7^a^	Nontidal, managed hydrology
East End (US‐Tw4)	Crops	2013/11	323	Planted sections and seeding	100	Nontidal, managed hydrology
Mayberry (US‐Myb)	Pasture and pepperweed	2010/14	121	Margins planted	82.4	Nontidal, managed hydrology
West Ponds (US‐Tw1)	Crops	1997/11	3	Planted sections	100	Nontidal, managed hydrology

^a^ Proportions calculated based on 2018 images to reflect last monitoring year conditions in Sherman Wetland.

#### 2.1.1 Non‐Tidal Wetlands

The East End (Lat: 38.1027, Long: −121.6413, AmeriFlux ID: US‐Tw4) and West Pond (Lat: 38.1074, Long: −121.6469, Ameriflux ID: US‐Tw1) wetlands are located on Twitchell Island. West Pond is the oldest wetland among our case studies, which was constructed in 1997 as a pilot wetland to study the potential of peat accumulation for subsidence reversal. The site is fully vegetated, with a dense canopy, and during restoration works, some of the sections were initially planted with tules. West Pond is the only site in this study where measurements did not start until several years after reconstruction work and therefore is missing data from the initial post‐restoration period. East End wetland was constructed in 2013 from a previous cornfield. East End was partially planted (along the surrounding levees) with stems and rhizomes of tule and cattail. While initially it had a mixture of open water and vegetated areas, it has reached full coverage and dense vegetation in 2024. Due to the proximity of both sites, they experience similar climatic conditions, with a mean annual precipitation of 421 mm and a mean annual temperature of 15.6°C over the whole measurement period. More background information on sites can be found in Hemes et al. (2019); Miller and Fujii (2011); Miller et al. (2008).

Two other, non‐tidal restored wetlands are located on Sherman Island. Mayberry Wetland (Lat: 38.0499, Long: −121.7650, AmeriFlux ID: US‐Myb) was restored in 2010 from an annual grassland pasture. The wetland was designed to be more heterogeneous than those in Twitchell Island, and varying bathymetric modifications created a complex mosaic of deep (up to 2 m in depth) and shallow open water areas, channels, and ponds. Only the margins of the wetland were planted with cattails and tules. The site's vegetation is currently dominated by tule, cattail, and on drier edges with *Sorghum halepense* . With a similar, heterogeneous design, Sherman Wetland (Lat: 38.0369, Long: −121.7547, US‐Sne) was restored from a pasture in 2016. No initial planting was done, and the vegetation is still developing almost 10 years later. Both sites experience similar mean annual air temperatures of 16.1°C and 311 mm of precipitation. As all these non‐tidal wetlands are below sea level, they lack natural hydrologic connectivity and depend on a pump system to actively manage water levels and maintain inundation suitable for marsh vegetation.

#### 2.1.2 Tidal Wetlands

The Gilbert Tract wetland (Lat: 38.0015, Long: −121.6691, AmeriFlux ID: US‐Dmg), located on Dutch Slough Marsh, is a tidal wetland that was restored in 2018. The restoration work was carried out by the California Department of Water Resources, with active revegetation starting in late 2019, followed by tidal action restoration in October 2021. Hydrological reconnection was achieved through several levee breaches, targeted levee degradations, and raises. Marsh plain elevations were reconstructed by grading each parcel to the appropriate tidal heights. Existing tule clumps were salvaged during construction and relocated to managed planting areas to promote vegetation spread. Riparian habitat was restored by planting native woody and herbaceous species, followed by weed control and the seeding of native grasses, sedges, and wildflowers at suitable elevations. The dominant vegetation at this site is cattail and tule, with open water patches covered with mosquito fern (*Azolla* spp.). More information about the restoration plans can be found at the California Department of Water Resources webpage (www.water.ca.gov). The site has an annual precipitation of 365 mm with a mean annual temperature of 16.1°C. The Hill Slough wetland (Lat: 38.2368, Long: −122.0212, AmeriFlux ID: US‐Hsm), located in the Suisun Marsh, is another tidal wetland that underwent tidal restoration in autumn 2021. Restoration works included levee breaching and levee lowering. No grading or active replanting was conducted in the study area. The primary goal was to restore tidal hydrology, vegetation, and improve habitat conditions for wildlife. After 3 years of tidal action restoration, the site is still mostly open water, with few small patches of tule, which cover less than 5% of the total wetland area. The mean annual temperature is 15.6°C and annual precipitation of 450 mm.

### 2.2 Eddy Covariance Measurements

We used the EC technique to continuously measure exchanges of CO_2_, CH_4_, H_2_O, and energy between the wetland ecosystems and the atmosphere (Baldocchi et al. 1988). Fluxes were measured using open‐path infrared gas analyzers (LI‐7500 or LI‐7500A for CO_2_ and H_2_O, and LI‐7700 for CH_4_; LI‐COR Inc., Lincoln, NE, USA). Sonic anemometers (WindMaster Pro 1352 or 1590, Gill Instruments Ltd., UK) recorded three‐dimensional wind vectors and speed of sound at 20 Hz. Fluxes were calculated from the 30‐min covariance between vertical wind speed and each scalar, following established correction procedures (Hatala et al. 2012; Hemes et al. 2019; Knox et al. 2015). Then, we performed a 2D coordinate rotation to minimize tilt errors and force the mean vertical velocity to zero (Wilczak et al. 2001). Additionally, we applied quality control filters for atmospheric stability, turbulence, friction velocity, wind direction, signal spikes, variances, covariances, and sensor performance, removing non‐ideal data accordingly. To estimate annual carbon and GHG budgets, we gap‐filled missing fluxes using artificial neural networks (ANNs) trained on 30‐min averaged meteorological data specific to each site. Inputs included air temperature, net radiation, photosynthetically active radiation, vapor pressure deficit, water level, green chromatic coordinate, wind direction, and friction velocity (Eichelmann et al. 2018; Knox et al. 2015; Moffat et al. 2007; Papale et al. 2006). Training, validation, and testing datasets were drawn from k‐means clusters to minimize seasonal and diel bias. We selected the simplest ANN architecture for which further complexity reduced the mean root square error by less than 5%. This process was repeated 20 times, and the median prediction across runs was used for gap‐filling. Net ecosystem exchange (NEE) of CO_2_ was partitioned into ecosystem respiration (ER) and gross primary productivity (GPP) using ANNs, which predicted daytime ER based on nighttime observations when photosynthesis was inactive. Negative NEE values indicate net CO_2_ uptake by the ecosystem.

### 2.3 Flux Footprints and Vegetation Monitoring

To describe the spatial extent of flux densities measured by EC at a height above the canopy, we used a two‐dimensional footprint model following (Kljun et al. 2015). Under the assumption of horizontally homogeneous turbulence, we defined the vertical turbulent flux, Fc (kg m^−2^ s^−1^), within the local footprint coordinate system as:

$$F_{C}\left(0,0,Z_{m}\right)=\int_{0}^{+\infty }\int_{-\infty }^{+\infty }Q\left(x,y\right)f\left(x,y,Z_{m}\right)\text{dxdy}$$

where $Z_{m}$ is the measurement height, Q is the surface flux (kg m^−2^ s^−1^), and f is the footprint function (m^−2^). Note that since f is always specific to $Z_{m}$, therefore, the vertical reference in function was ignored, for simplicity. Then, the total two‐dimensional flux footprint was expressed as the product of a crosswind‐integrated footprint function ($f^{y}$) and a crosswind dispersion function ($D_{y}$) (Horst and Weil 1992; Rey‐Sanchez et al. 2022):

$$f\left(x,y\right)=f^{y}\left(x\right)\;D_{y}\left(x,y\right)$$

The parameterization for the derivation and evaluation of $f^{y}$ and $D_{y}$ was based on a backward Lagrangian stochastic particle dispersion model (see (Kljun et al. 2002) for more details). In short, this modeling scheme is suitable for our wetland sites as it captures a range of atmospheric stability (i.e., unstable to stable stratifications) and surface characteristics. After applying these above functions, we ran the footprint model at a spatial resolution of 3 m, where the EC tower was centered at the origin (0,0) with weight of the footprint distributed for each of the cells in the grid.

In this study, we calculated and used the maximum footprint contour of 80% across the sites within the month of August 2020 (Sherman Wetland) and 2023 (West Pond, East End, Mayberry, Gilbert Tract, and Hill Slough) for the peak growing season.

We further computed a remote sensing‐based indicator of vegetation cover within the footprint as Green Normalized Difference Vegetation Index (GNDVI; Gitelson et al. 1996) using green and near‐infrared bands of Landsat satellite Level 2, Tier 1 surface reflectance products. Landsat vegetation indices have shown strong correlations with ground‐measured wetland GPP in this region in earlier research (e.g., Dronova et al. 2021; Knox et al. 2017). To focus on the peak green biomass season, we used all available Landsat imagery for the months of August and September and mosaicked them into a single image representing maximum greenness for each year since 2010. Using spatial boundaries of the respective footprint contours, we then computed mean values of GNDVI for each footprint in each year.

### 2.4 Radiative Balance and Radiative Forcing

We calculated the radiative forcing of wetland restoration actions using an atmospheric perturbation model as described in (Arias‐Ortiz et al. 2021; Bridgham et al. 2014; Neubauer 2014; Neubauer and Megonigal 2015). The model simulates the atmospheric inventories of ecosystem‐derived CO_2_ and CH_4_ by accounting for emissions or uptake, and first‐order removal processes. CH_4_ follows simple decay‐based dynamics, while CO_2_ is buffered through exchanges with multiple non‐atmospheric reservoirs over different timescales. CO_2_ dynamics were modeled using five non‐interacting pools with distinct lifetimes, reflecting short‐ to long‐term processes (Arias‐Ortiz et al. 2021). Different radiative efficiencies and atmospheric residence times of CO_2_ and CH_4_ were considered, as well as the oxidation of CH_4_ to CO_2_. The CO_2_ and CH_4_ pools were converted to kg CO_2_ and kg CH_4_, respectively, before applying radiative efficiency values. The radiative balance of restored wetlands was modeled for a 200‐year period under stable environmental conditions considering (1) the net radiative effect of CH_4_ emissions and CO_2_ uptake emitted as a pulse (instantaneous radiative balance) and (2) the net cumulative effect of CH_4_ and CO_2_ dynamics of sustained emissions and uptake year after year (cumulative radiative balance). We used CH_4_ emissions and NEE from EC measurements as input variables.

The radiative forcing caused by wetland restoration was calculated as the difference between the radiative balance of the restored wetland and that of the previous land use (Arias‐Ortiz et al. 2021; Neubauer and Verhoeven 2019), which was corn for East End and pasture for Mayberry, Sherman Wetland, and Gilbert Tract based on Hemes et al. (2019). Before tidal restoration, Hill Slough was a seasonal brackish wetland that was dry in summer, and therefore we assume the CH_4_ before restoration was negligible. Greenhouse gas emissions and sequestration from crop and pasture in the Delta have been estimated by Hemes et al. (2019) using EC flux systems. Specifically, we used published data from Twitchell corn and Sherman pasture as they are in the same Delta Islands as our managed wetlands and would have similar soil carbon and characteristics to pre‐restoration land uses.

The wetland switchover time (i.e., the time needed to switch from a positive to a neutral or negative radiative forcing) was determined as the crossover point where net radiative forcing reaches zero. This occurs when the warming effect due to CH_4_ emissions is overtaken by the cumulative removal (i.e., cooling effect) of CO_2_. We run a Monte Carlo simulation (*n* = 1000) to capture the variability in switchover times due to the interannual variability in CH_4_ emissions and NEE.

### 2.5 Soil Sampling and Soil Chemical Analyses

Soil samples for C% and N% from Delta wetlands were collected and analyzed on several occasions from the top‐soil layer (0–15 cm). In August 2018 from West Pond (*n* = 10), Mayberry (*n* = 10), East End (*n* = 10), Sherman Wetland (*n* = 10); in December 2023 from Gilbert Tract (*n* = 19) and Mayberry (*n* = 21), in August 2024 from Gilbert Tract (*n* = 15) and Mayberry (*n* = 16), and in May 2025 from Hill Slough (*n* = 10). For 2024 and 2025 sample analyses we used CHN MACRO cube element analyzer (Elementar GmbH, Germany) and for 2018 samples CE Elantech analyzer (Lakewood, NJ, USA).

### 2.6 Statistical Analyses

We applied k‐means clustering to explore patterns across all wetland sites based on annual cumulative NEE, annual CH_4_ fluxes, and wetland age as an input. The number of clusters was pre‐defined, and the algorithm grouped sites by minimizing within‐cluster variance. To visualize cluster separation and structure, we applied principal component analysis (PCA) to reduce the dataset to two dimensions. For uncertainty analysis of the gap‐filled data, we calculated the proportion of missing (i.e., gap‐filled) records for each year. Annual uncertainty was estimated by multiplying the cumulative flux by the fraction of gap‐filled records relative to the total number of records in that year, assuming that uncertainty is proportional to the extent of missing data. To determine CO_2_ uptake length, we identified the first day in each calendar year when the 10‐day moving average of NEE dropped below zero (considered the start of the uptake period), and the day it became positive again (end of uptake). Only full calendar years were included in the analysis. To assess differences in CO_2_ uptake length among sites, we used the non‐parametric Kruskal‐Wallis test. All statistical analyses were conducted in R using the following packages: openair (Carslaw and Ropkins 2012), ggplot2 (Wickham 2009), factoextra (Kassambara and Mundt 2020), and dplyr (Wickham et al. 2023).

To examine the relationships between fluxes and environmental drivers, we applied mutual information (MI) analysis at the three mature sites: West Pond, East End, and Mayberry. MI is particularly suitable for detecting both linear and nonlinear associations without assuming normality or specific distributional forms (Ahmadi et al. 2022). The MI value quantifies dependency between two variables: it is zero if they are independent and increases with stronger dependence. We computed MI using a nonparametric method based on entropy estimation from k‐nearest neighbors, as described by (Kraskov et al. 2004) and (Ross 2014). Prior to analysis, we conducted complete‐case filtering to remove rows with missing data. Only measured (non‐gap‐filled) data were used to ensure that results reflected observed relationships. For each flux variable (NEE and CH_4_), MI was computed against the following daily environmental variables: air pressure (PA), photosynthetically active radiation (PAR), air temperature (TA), soil temperature at 2 cm depth (TS), and water table (WT). We chose these variables as PAR drives GPP and NEE = GPP—ER. TA affects kinetics of GPP and influences TS, which affects soil respiration but also methanogenesis. Air pressure, on the other hand, affects gas exchange as well as the evolution of CH_4_ emissions, while WT affects CH_4_ production and inhibits soil respiration due to inundation and anaerobic conditions. MI values were normalized to facilitate comparison across variables. All MI computations were performed using the Scikit‐learn library in Python.

## 3 Results

**FIGURE 2:**
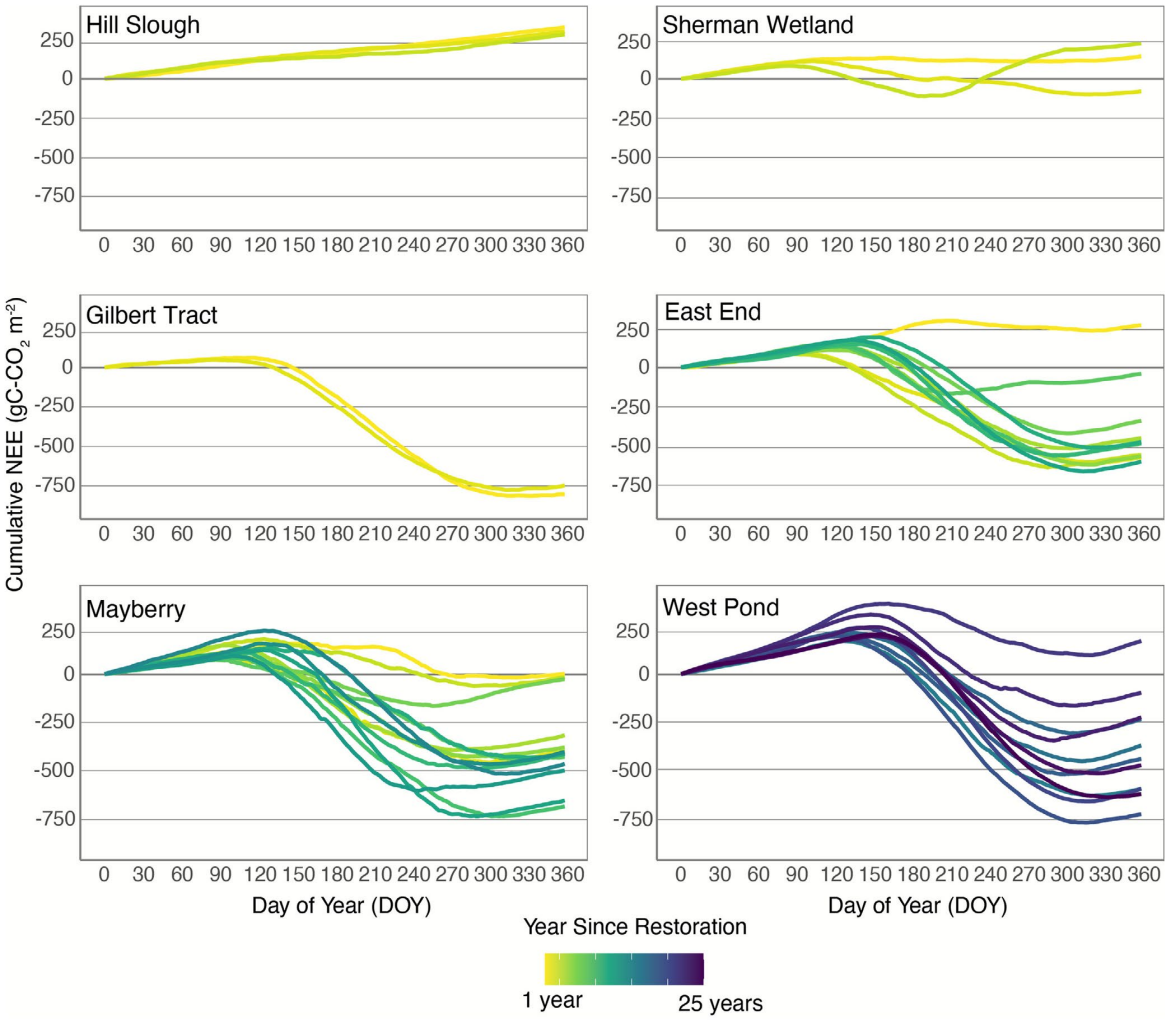
Cumulative annual net ecosystem exchange (NEE) (gC m^−2^) throughout the year for all site‐years.

**FIGURE 3:**
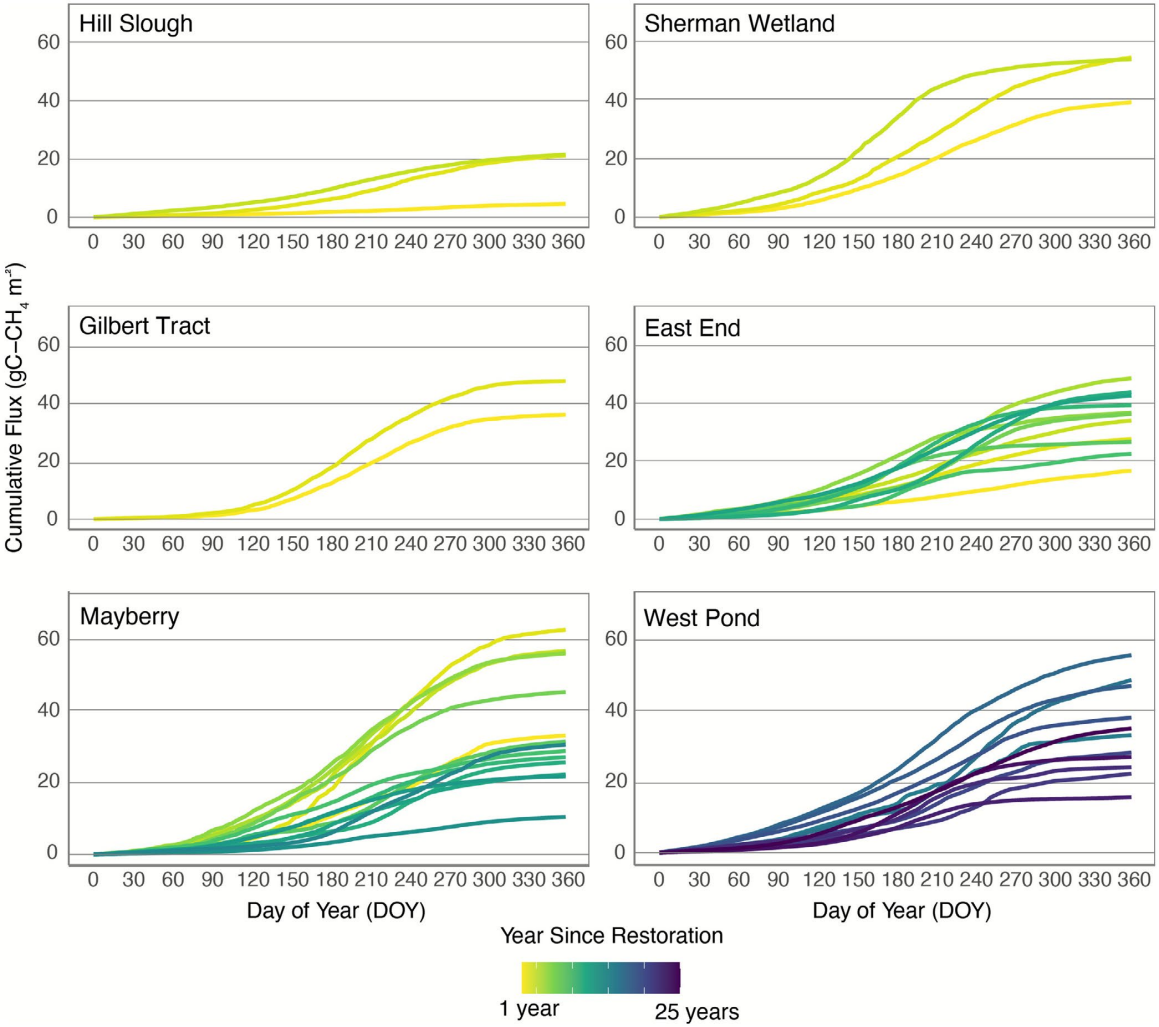
Cumulative annual methane flux (gC m^−2^) throughout the years for all site‐years.

All restored wetlands in the Delta exhibited seasonal and interannual variations in both CO_2_ (Figure 2) and CH_4_ fluxes (Figure 3). During the growing seasons, NEE was negative, indicating net CO_2_ uptake, whereas during the winter months, NEE was positive due to net respiration caused by dormant, deciduous vegetation. All sites showed an immediate increase in CH_4_ emissions following restoration, primarily driven by flooding. This was accompanied by a clear seasonal pattern, with CH_4_ fluxes peaking in summer and dropping significantly during the winter months.

### 3.1 Interannual Carbon Flux Variability

Each wetland displayed distinct post‐restoration CO_2_ patterns (Figure 2). Notably, only Gilbert Tract Wetland became a significant CO_2_ sink within the first year after restoration, with a cumulative NEE of −801 ± 184 gC m^−2^ year^−1^ (mean ± annual uncertainty). In contrast, East End and Mayberry functioned as either small CO_2_ sources or near‐neutral in their first year post‐restoration, with a mean positive NEE of 142 ± 64 gC m^−2^ year^−1^ across three site‐years. However, when excluding the immediate post‐restoration year, both Mayberry and East End became consistent CO_2_ sinks, with mean uptake values of −449 ± 188 gC m^−2^ year^−1^ and −451 ± 168 gC m^−2^ year^−1^, respectively. Hill Slough and Sherman Wetland, on the other hand, remained a continuous CO_2_ source even 3 years after restoration, with a 3‐year average cumulative NEE of 310 ± 154 gC m^−2^ year^−1^. West Pond monitoring started at 14 years post‐restoration, so its initial trajectory is unknown. However, its overall mean CO_2_ uptake was −372 ± 261 gC m^−2^ year^−1^, with a maximum uptake of −721 ± 491 gC m^−2^ year^−1^ in 2017.

**FIGURE 4:**
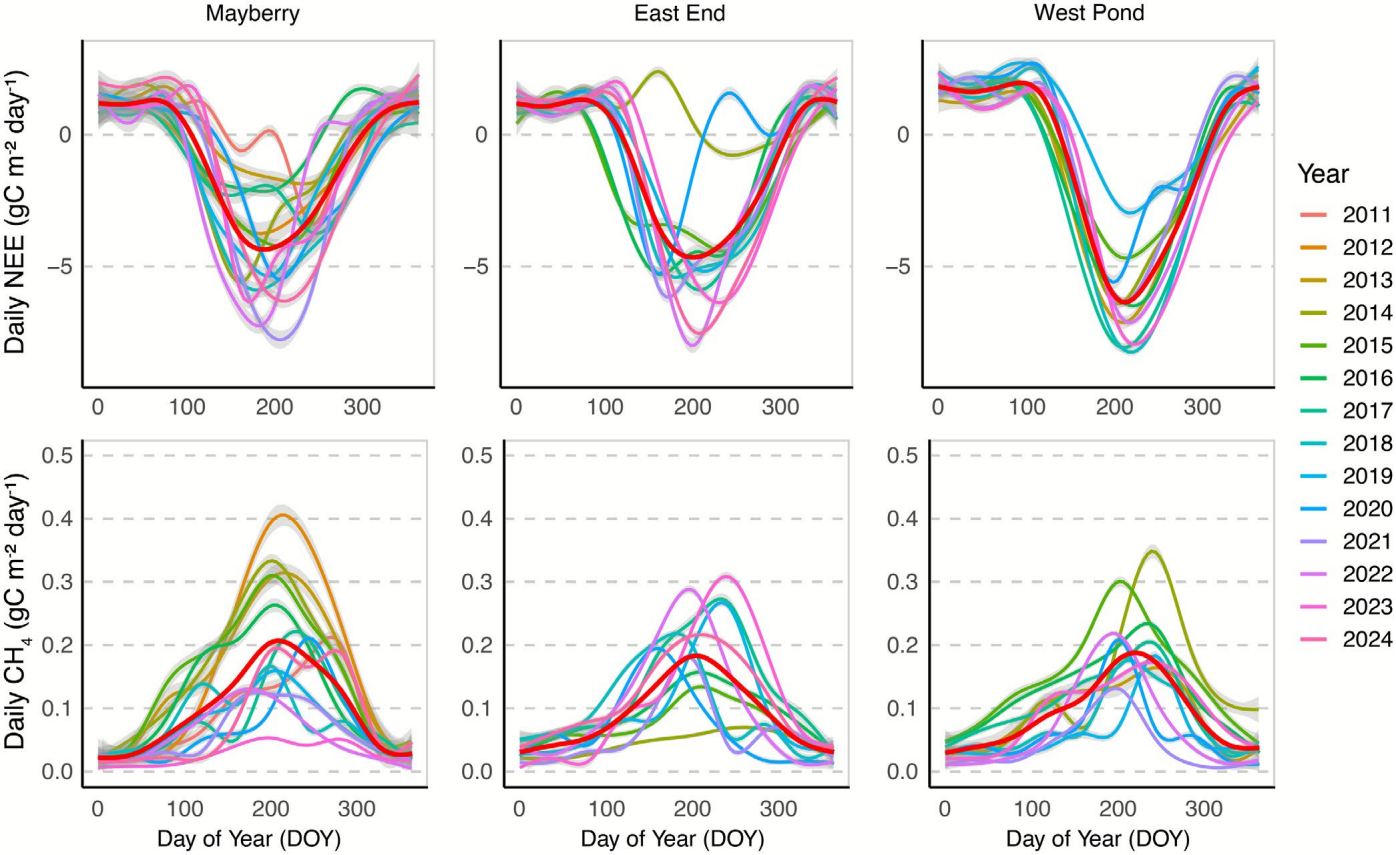
Ten day moving average of annual NEE and CH_4_ dynamics over Mayberry, East End, and West Pond wetlands. The red line represents the multi‐year mean of daily fluxes at each site.

Seasonal dynamics of daily NEE dynamics at the mature wetlands in East End, Mayberry, and West Pond showed strong carbon uptake during the summer months (Figure 4), with the sites transitioning to net carbon sources from late autumn through early spring. While East End and West Pond exhibited relatively stable interannual patterns, Mayberry showed high variability in CO_2_ exchange throughout the study period.

The average annual CH_4_ flux across all six wetlands was 35 ± 15 gC m^−2^ year^−1^, based on 44 site‐years of data (Figure 3). Notably, while post‐restoration NEE trajectories were near neutral or small CO_2_ sources, CH_4_ emissions increased substantially following flooding. At East End, cumulative CH_4_ emissions increased over the first four years, from 16 ± 7 to 50 ± 20 gC m^−2^ year^−1^, then declined for four consecutive years to nearly post‐restoration levels, before increasing again. Eleven years after restoration, emissions reached 43 ± 17 gC m^−2^ year^−1^. A similar trend was observed at Mayberry, where emissions initially increased from 34 ± 15 gC m^−2^ year^−1^ after restoration to 57 ± 23 gC m^−2^ year^−1^. By the sixth year, CH_4_ emissions began decreasing substantially, reaching their lowest level 13 years after restoration with 11 ± 2 gC m^−2^ year^−1^. However, the following year, emissions tripled to 31 ± 5 gC m^−2^ year^−1^. West Pond exhibited similar fluctuating trends, with CH_4_ emissions peaking at 56 ± 33 gC m^−2^ year^−1^ at year 17 post‐restoration, then decreasing to 16 ± 9 gC m^−2^ year^−1^ by year 23, followed by an increase to 35 ± 24 gC m^−2^ year^−1^ at year 25 years post restoration.

Despite seasonal trends of summer CH_4_ emission peaks and winter declines, the magnitude and timing of these peaks varied substantially from year to year across sites (Figure 4). This variability was particularly pronounced in daily CH_4_ fluxes, which exhibited distinct and inconsistent patterns among wetlands.

**FIGURE 5:**
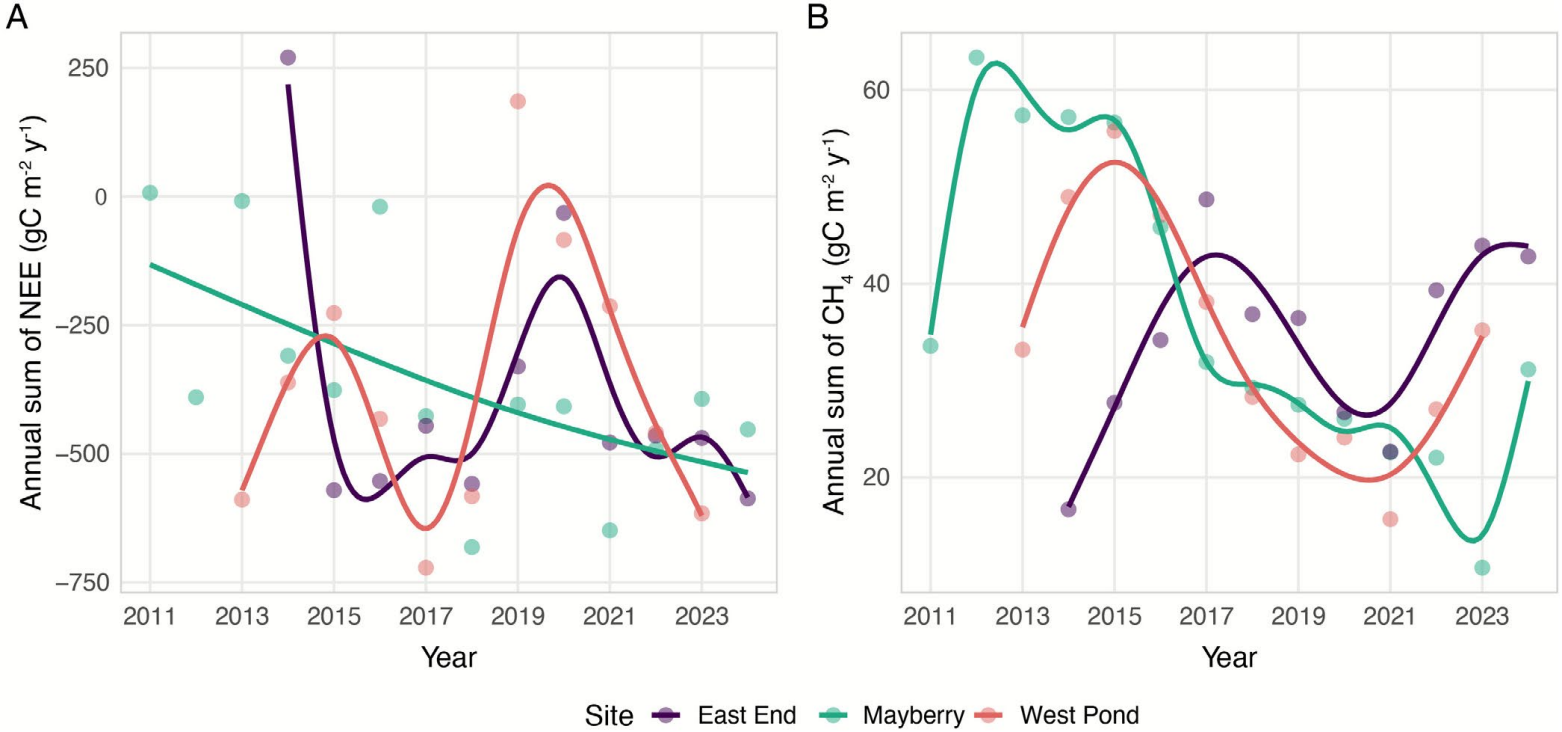
Interannual variation in cumulative NEE (A) and CH_4_ (B) fluxes with a loess smoothing fit across all years for East End, Mayberry, and West Pond.

Mature wetlands (i.e., West Pond, East End and Mayberry) exhibited substantial site‐specific disturbances throughout the measurement periods that affected the annual CO_2_ uptake (Figure 5A) and CH_4_ emissions (Figure 5B). Across East End and West Pond, the most impactful event occurred in 2020, where a major water level drawdown led to a sharp decline in ecosystem CO_2_ uptake and one of the lowest annual CH_4_ emissions on record (Figure 5B). Similarly, Mayberry experienced three major disruptions: an insect infestation in 2013 and 2016, and salinity stress from saltwater intrusion during 2015–2016, both of which reduced CO_2_ uptake to near zero (Figure 5A). In 2019, West Pond temporarily became a net CO_2_ source due to water drawdown and vegetation harvesting linked to a separate experiment. Water level drawdown also affected the East End in 2019, where a sharp decline in CO_2_ uptake was visible (Figure 5A). When focusing on the mature phase, excluding early post‐restoration and disturbance years (e.g., insect infestation), the average annual CO_2_ uptake across Mayberry, West Pond, and East End was −449 ± 173 gC m^−2^ year^−1^ (mean ± SD; 30 site‐years), underscoring the influence of both recovery trajectories and disturbance events on carbon dynamics.

### 3.2 Wetland Age, Vegetation, and Soil Development

**FIGURE 6:**
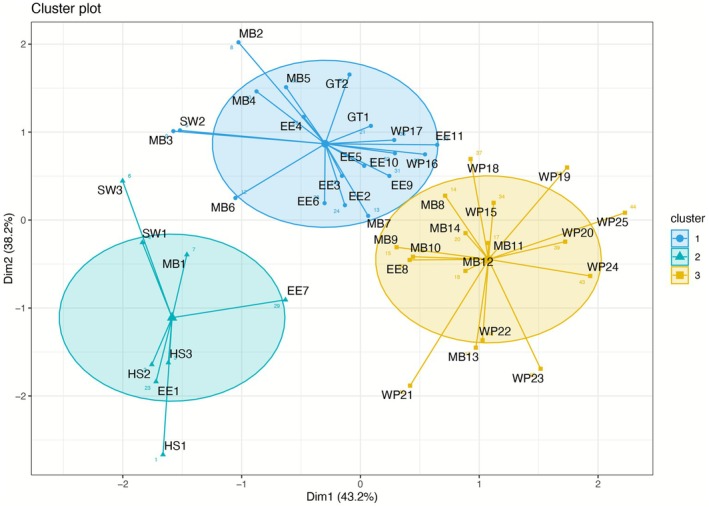
Clustering the cumulative NEE and CH_4_ fluxes by the age of the wetland since restoration. MB—Mayberry, SW—Sherman Wetland, EE—East End, WP—West Pond, GT—Gilbert Tract, HS—Hill Slough. The number in each site indicates year since restoration.

The cluster analyses clearly reveal three distinct clusters and that site‐years of young wetlands, with a mean age of 2 years, have a separate cluster (Figure 6; Cluster 2). Well‐advanced, partially to fully vegetated wetlands, with an average age of 6 years, fall into Cluster 1, while mature wetlands with a mean age of 15 years form the third cluster. One exception is the Gilbert Tract wetland, which, despite being only 3 years old, falls into Cluster 1 altogether with well advanced sites. Few other exceptions due to severe site‐specific disturbances like East End at Year 7 (Figure 6, EE7) and West Pond at years 16 and 17 (Figure 6, WP16 and WP17) fell into Cluster 2 and Cluster 1, respectively.

**FIGURE 7:**
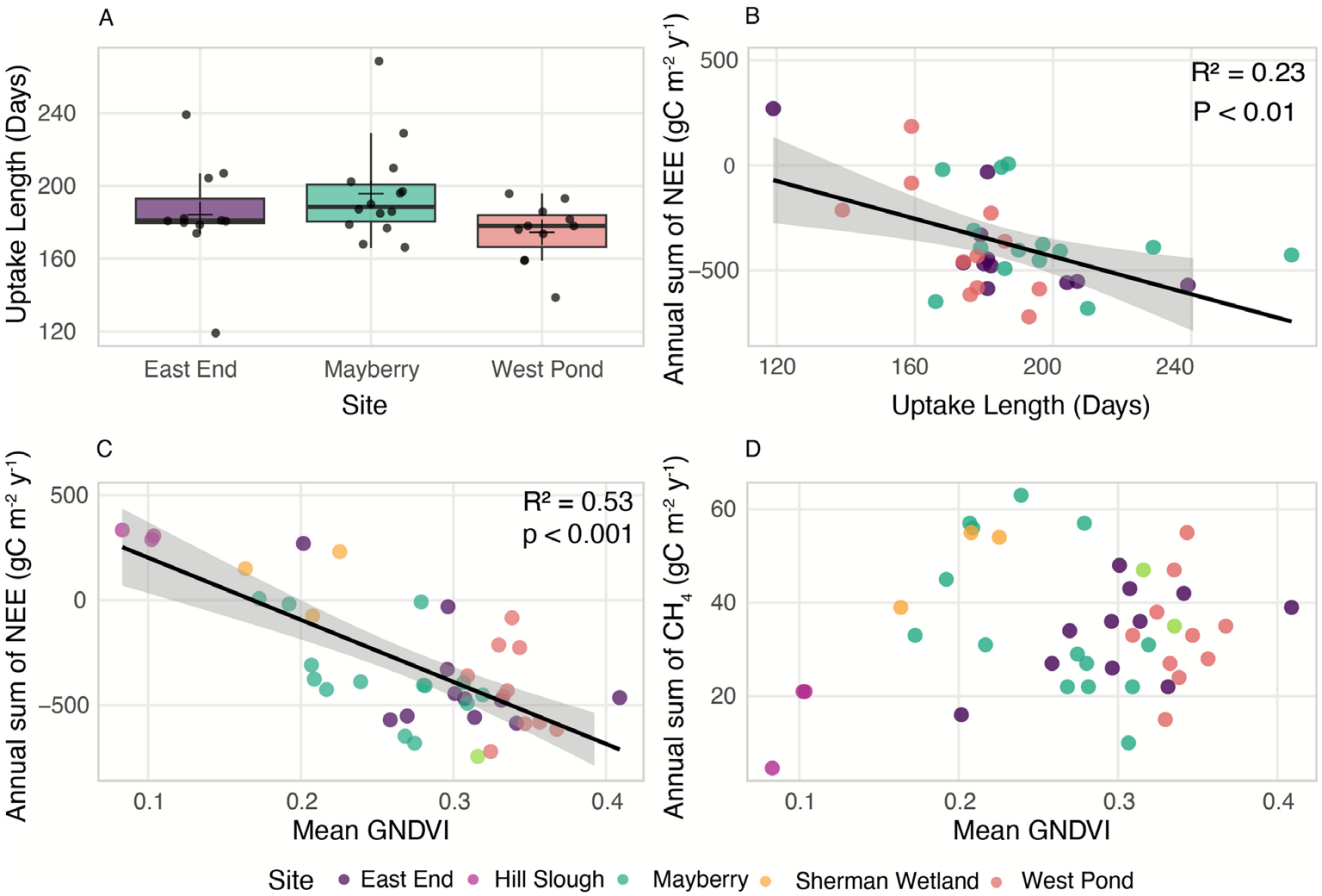
Relationships between vegetation metrics and carbon fluxes across site‐years at East End, Mayberry, West Pond, Hill Slough, and Gilbert Tract wetlands. (A) Uptake length (days), (B) cumulative annual NEE as a function of uptake length, (C) cumulative annual NEE vs. mean Green NDVI (GNDVI), and (D) cumulative annual CH_4_ flux vs. mean GNDVI.

When considering the uptake season length, the mean uptake length (± standard error) in East End, Mayberry, and West Pond was 184 ± 9, 196 ± 7, and 175 ± 5 days, respectively (Figure 7A). Based on the Kruskal‐Wallis test, there were slight but not statistically significant differences (*p* = 0.08) between the sites. On the other hand, the uptake length showed a statistically significant negative correlation with cumulative annual NEE (*R* ^2^ = 0.23, *p* < 0.01; Figure 7B), indicating that longer uptake lengths are typically associated with higher CO_2_ sequestration. Additionally, the mean GNDVI value in the footprint had a strong negative correlation with NEE (*R* ^2^ = 0.53, *p* < 0.001; Figure 7C), suggesting that higher vegetation cover in the footprint led to greater uptake of the total NEE. No correlation was found between mean GNDVI and CH_4_ flux (Figure 7D).

**FIGURE 8:**
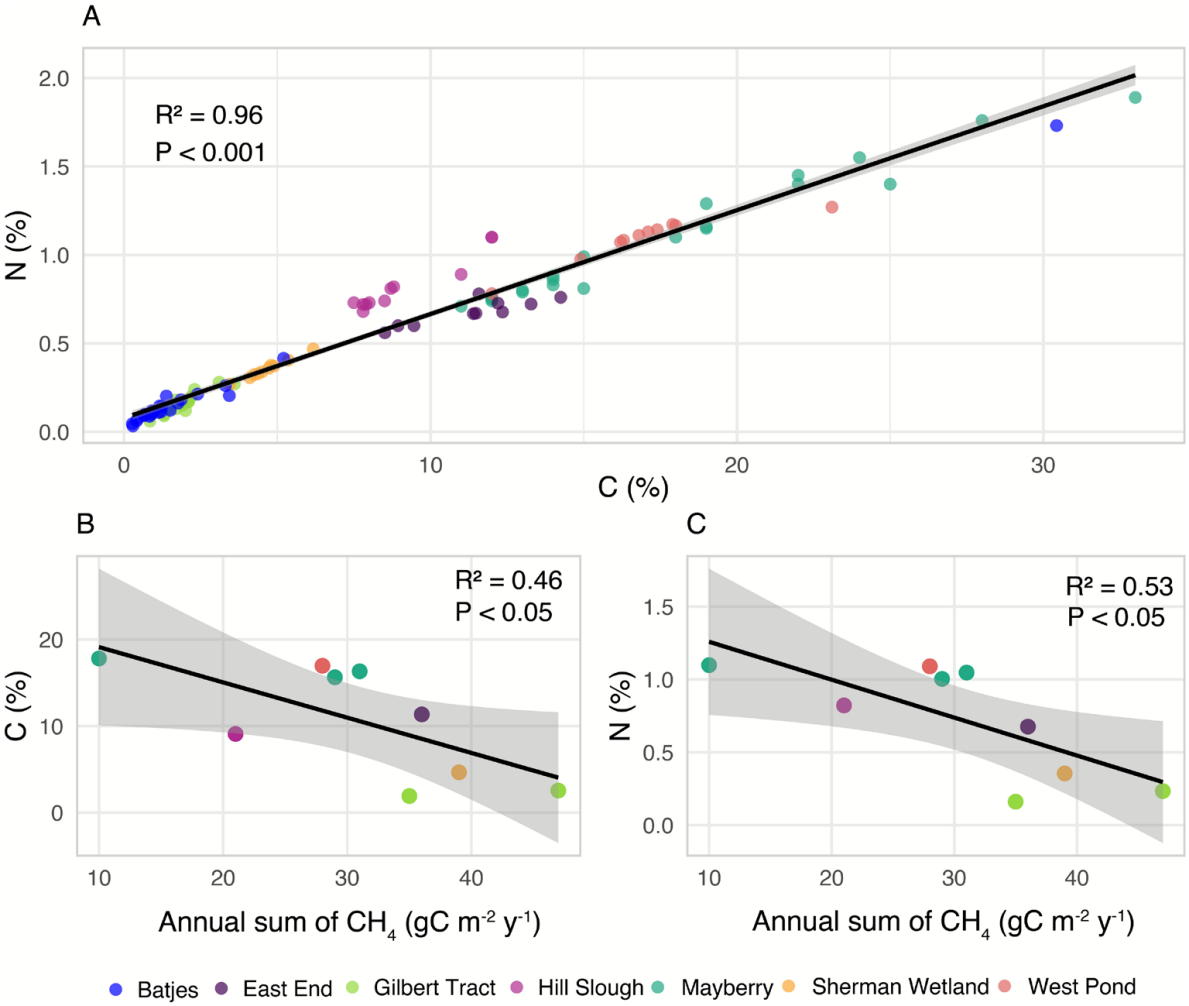
Soil C% and N% content in the Delta restored wetlands and world soils based on Batjes (2014) (A) and the correlation plots between C% (B), N% (C), and annual CH_4_ emission across different wetlands.

Soil carbon and nitrogen concentrations varied strongly across the study sites, reflecting differences in land use history and wetland age (Figure 8). The highest mean C% and N% were observed at West Pond (16.9% ± 2.8%, 1.09% ± 0.13%), Mayberry (16.8% ± 4.8%, 1.06% ± 0.29%), and East End (11.3% ± 1.9%, 0.68% ± 0.07%), which represent the oldest restored wetlands. In contrast, the youngest wetlands, Hill Slough, Gilbert Tract, and Sherman Wetland had significantly lower C and N concentrations, averaging 9.1% ± 1.7% C/0.82% ± 0.15% N, 2.2% ± 0.8% C/0.19% ± 0.08% N, and 4.7% ± 0.7% C/0.35% ± 0.06% N, respectively. While absolute C and N concentrations varied considerably across sites, C:N ratios were more consistent, ranging from 11.1 to 16.7, with slightly lower values in the youngest sites. Batjes World Soils, used here as a global reference dataset (Batjes 2014), fell mostly at the lower end of the range, except for histosol (C% = 30.4% and N% = 1.7%) that are on the upper end. A strong correlation between C and N content was observed across all sites (*R* ^2^ = 0.97, *p* < 0.001; Figure 8A), and both variables increased with wetland age, highlighting progressive organic matter accumulation in older wetlands. We examined relationships between mean soil C% and N% (based on 10 sampling locations per wetland) and annual CH_4_ and NEE for Hill Slough (2024), Gilbert Tract (2023–2024), Mayberry (2018, 2023–2024), East End (2018), West Pond (2018), and Sherman Wetland (2018). Surprisingly, total annual CH_4_ emission showed moderate but significant negative correlations with soil C% (*R* ^2^ = 0.46; Figure 8B) and N% (*R* ^2^ = 0.53; Figure 8C), whereas NEE exhibited no detectable relationship with either soil parameter.

### 3.3 Relative Importance of Environmental Drivers of NEE, CH_4_ , and Related Variability

**FIGURE 9:**
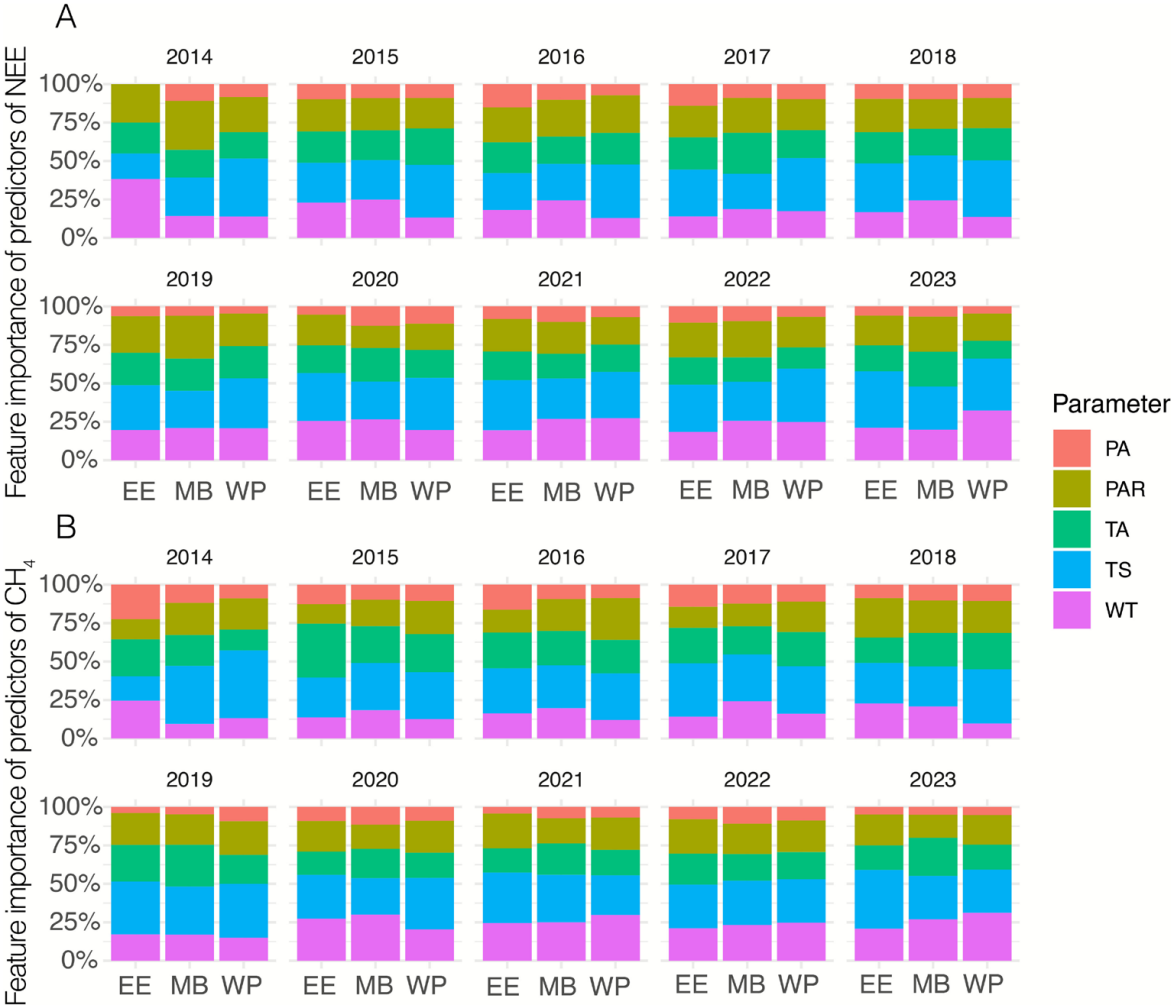
Feature importance of predictors of NEE (A) and CH_4_ (B), including air pressure (PA), photosynthetically active radiation (PAR), air temperature (TA), soil temperature at 2 cm (TS), water table depth (WT) in East End (EE), Mayberry (MB), and West Pond (WP) across the entire year.

MI analyses over non‐gap filled 30 min average flux revealed distinct patterns in the relative importance of environmental drivers across the three mature wetlands (East End, Mayberry, and West Pond) over the 10‐year observation period (2014–2023) (Figure 9). WT consistently emerged as a dominant control across all sites, accounting for approximately 10%–30% of relative importance for both NEE (Figure 9a) and CH_4_ (Figure 9b). Moreover, the feature importance of WT to predict CH_4_ emissions increased over the years in all sites. For NEE, TS demonstrated substantial influence (approximately 20%–30% importance) across all sites, particularly at Mayberry where it frequently represented the second most important factor after water table depth. While TS in Mayberry and West Pond wetland remained almost in the same range throughout the analysis period, the importance of TS for East End NEE became stronger each year after restoration. Theil‐Sen trend analyses (data not shown) also revealed that TS has a declining trend (−0.44°C year^−1^, *p* < 0.001) at East End, while no trend was observable in other sites. For CH_4_, TS was the most important factor across all sites with the mean importance over the years between 29% and 32%. The relative importance of TA and PAR showed more variability between sites but generally maintained moderate influence (10%–20%) throughout the study period for both NEE and CH_4_ (Figure 9a,b). PA typically showed lower relative importance.

On site level, East End exhibited the highest sensitivity to water table fluctuations, with WT consistently accounting for 20%–30% of relative importance throughout the decade for NEE. Soil temperature and air temperature showed comparable levels of importance (15%–20% each). While for CH_4_, the TS had highest importance, showing also clear increasing trend over 10 years from 16% to 38%. Mayberry displayed a more balanced distribution of feature importance for NEE, with TS frequently matching or exceeding the importance of WT (both approximately 20%–25%). This site also showed the highest relative importance for TA among the three sites, particularly during 2016–2018, when it accounted for nearly 20% of the explained variance. In terms of CH_4_, the TS showed the highest relative importance, which was in a similar range over the years, being between 24% and 38%. For NEE, West Pond demonstrated the most distinctive pattern, with consistently higher importance for TS (25%–30%) compared to the other sites. WT, while still important (approximately 15%–20%), showed less dominance here than at the other sites. In terms of CH_4_, the importance of WT increases rapidly over the years from 10% to 31% while relative importance of all other parameters showed slightly decreasing trend.

### 3.4 Long Term Climatic Impact of Wetland Restoration

**FIGURE 10:**
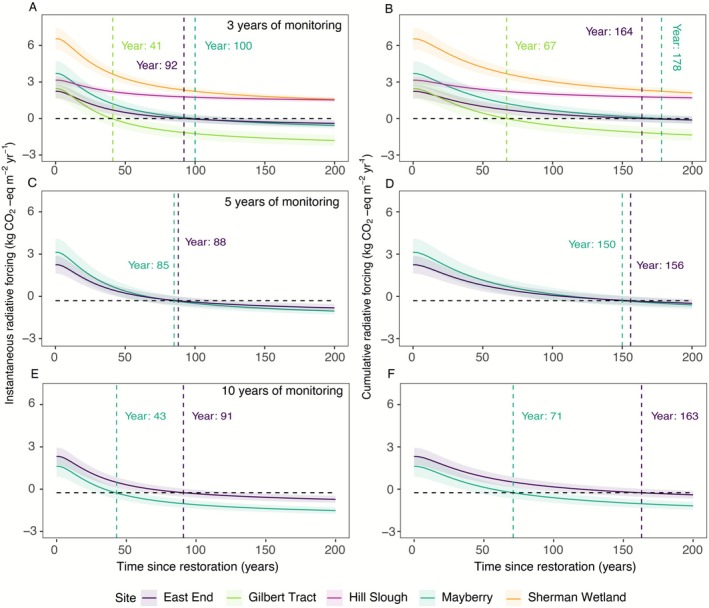
Instantaneous and cumulative forcing of net CO_2_‐equivalent greenhouse gas fluxes based on 3 years of monitoring data (A and B), 5 years (C and D), and 10 years (E and F). The Δ radiative forcing is calculated as the difference between the radiative balance of the restored wetlands and that of the previous land use (pasture for Mayberry, Sherman Wetland, Hill Slough, Gilbert Tract, and corn for East End). Net GHG fluxes were converted to CO_2_‐equivalents as the product of the gas flux and the ratio of the cumulative radiative efficiencies of a kg of CH_4_ and CO_2_ over time, after accounting for the indirect forcings of CH_4_. The shaded area represents 95% confidence in switchover times.

Figure 10 presents modeled trajectories of radiative forcing following wetland restoration across six study sites, based on data from 3, 5, and 10 years of monitoring. The approach to look at how different monitoring periods affect modeled trajectories was chosen to provide suggestions about the necessary monitoring duration of representative variability, which is an important question for restoration practitioners and policymakers who need reliable long‐term representative estimates of carbon benefits. We used two projection approaches, based on instantaneous pulsed emissions versus sustained emissions. The instantaneous scenario assumes the annual emissions occur as a single pulse that decays over time, whereas the cumulative scenario assumes emissions are sustained or ongoing year after year, all relative to CO_2_. The instantaneous radiative forcing (Figure 10, panels A, C, E) and cumulative radiative forcing (Figure 10, panels B, D, F) show distinct site‐specific patterns that evolve over time since restoration. Sherman Wetland and Hill Slough consistently demonstrated the highest initial positive radiative forcing and based on 200‐year time horizon, resulting in an undeterminable switchover time. This contrasts with Gilbert Tract, which exhibited negative radiative forcing values earliest among the sites, indicating a more rapid transition toward a climate benefit driven by a higher ratio of CO_2_ sequestration to CH_4_ emission. When comparing monitoring duration effects, the 3‐year monitoring projections (panels A, B) showed high variability between sites and wider uncertainty bands, particularly for Sherman Wetland and Hill Slough. The 5‐year monitoring projections (panels C, D) reveal more convergent trajectories in East End and Mayberry sites, with both sites projected to achieve net cooling effects (negative radiative forcing) within 85–88 years post‐restoration. The 10‐year monitoring data (panels E, F) further refined these projections, suggesting that Mayberry may reach the cooling threshold in as little as ~40 years, while East End would require approximately ~90 years. The cumulative radiative forcing projections (panels B, D, F) indicate that while all sites eventually trend toward negative values, the timeframes vary considerably are and unknown for young sites. Based on the 10‐year monitoring data (panel F), Mayberry is projected to reach net cooling by year 71, while East End would reach this milestone around year 163.

## 4 Discussion

Across three mature restored wetlands, each older than 10 years, the mean annual CO_2_ uptake was −449 ± 173 gC m^−2^ year^−1^, which is much larger than the global average of −153 ± 289 gC m^−2^ year^−1^ shown by (Baldocchi et al. 2018) over 1781 site years of published flux tower data. Therefore, Delta restored wetlands rank among the strongest CO_2_ sinks globally, with maximum annual CO_2_ uptake exceeding 800 gC m^−2^ year^−1^ (Fortuniak et al. 2017; Lund et al. 2010), which falls into the top 1% of the published data (Baldocchi 2020). In contrast, other land use types in the Delta exhibit high CO_2_ losses: corn and pasture lose CO_2_ at rates up to 826 and 306 gC m^−2^ year^−1^, respectively, while also emitting small amounts of CH_4_ (2 and 9 gC m^−2^ year^−1^) (Hemes et al. 2019). Alfalfa, due to active harvesting and high productivity, can act as a moderate CO_2_ sink (−396 gC m^−2^ year^−1^). Overall, CO_2_ uptake in Delta wetlands exceeds that of all other local land uses and is also substantially greater than that observed in other peat soil ecosystems such as natural boreal mires, where reported values range from −108 to −18 gC m^−2^ year^−1^ (Lund et al. 2010; Peichl et al. 2014), but lower than some in Poland, where annual uptake was between −560 and −980 gC m^−2^ year^−1^ (Fortuniak et al. 2017). In contrast, many restored peatlands continue to exhibit long‐term positive NEE, indicating persistent carbon losses to the atmosphere (Tong et al. 2025), like Hill Slough and Sherman Wetland. However, the high CO_2_ uptake in Delta restored wetlands is accompanied by elevated CH_4_ emissions. Annual CH_4_ fluxes can reach up to 60 gC m^−2^ year^−1^, with an average of 35 ± 14 gC m^−2^ year^−1^ based on 44 site‐years of measurements. In comparison, CH_4_ emissions from natural boreal and temperate peatlands are typically below 30 gC m^−2^ year^−1^ (Fortuniak et al. 2017; Tong et al. 2025). A global study over freshwater wetlands based on EC data showed that in most sites, annual CH_4_ emission rates ranged between 0–20 gC m^−2^ year^−1^ (Delwiche et al. 2021). Due to these above‐average CH_4_ emissions, the total radiative forcing prediction is key to understanding the beneficial context of restoration projects for high carbon sequestration over climate‐relevant time scales. On the other hand, these wetlands, even though they can be net GHG sources for decades, serve additional benefits such as soil build‐up, which is necessary in the Delta to stop subsidence and to protect the water system of California. These wetlands also provide many other ecological services such as habitat for waterfowl and fish, and food web for vertebrates and invertebrates.

### 4.1 Site‐Specific Factors, Management, and Disturbances Affecting the Greenhouse Gas Fluxes

Long‐term monitoring has shown that GHG exchange in wetlands is complex, that involves nonlinear asynchronous processes across time scales and site‐specific conditions are often more important than broader climatic trends in determining a wetland's annual carbon balance. While it might seem intuitive to expect a clear trajectory toward net cooling as wetlands become fully vegetated, the reality is far more complex. Even small, localized disturbances can drastically alter GHG uptake or release (Abbott et al. 2019). For instance, the Mayberry wetland experienced three significant disturbances over the years that affected the uptake. In 2013 and in 2016, there were insect infestations that affected the green aboveground biomass and a saltwater intrusion event from the second half of 2015 until mid‐2017 that stressed the vegetation, again reducing CO_2_ sequestration (Chamberlain et al. 2020). Saltwater intrusions may also have long‐term effects, particularly in systems with limited throughflow, where stressed vegetation can take years to recover (Herbert et al. 2015). We observed that significant water level drawdowns, such as those in the East End and West Pond wetlands in 2020, reduced CO_2_ uptake but also led to lower CH_4_ emissions. The negative impact of water level drawdown was so dramatic in East End that this particular year fell into cluster 2 altogether with recently restored wetlands (Figure 6). Similar outcomes have been reported in other studies, where drier soil conditions increased CO_2_ emissions while reducing CH_4_ emissions (Hassett et al. 2024; Okiti et al. 2025). When the water level remained stable, the West Pond wetland exhibited consistent CO_2_ uptake over the years without substantial fluctuations. Additionally, site level factors can also affect the occurrences of hot spots and hot moments for CH_4_ flux, which can also complicate the site‐level budgets (Okiti et al. 2025). The study by Rey‐Sanchez et al. (2025) showed that CH_4_ hot spots in Sherman Wetland were primarily driven by lower water levels relative to the soil surface, which enhanced ebullition and reduced CH_4_ oxidation. The formation of the hot spot was also partly attributed to restoration‐related disturbance, particularly the excavation of soil surface layers during levee construction, which likely reduced the thickness of the oxidized peat layer and facilitated CH_4_ transport from deeper soil.

### 4.2 Vegetation and Soil Development

Vegetation plays a critical role in CO_2_ uptake efficiency, with densely vegetated areas generally being more consistent at sequestering carbon. However, the accumulation of dead litter resulting in a dense legacy vegetation canopy, which was seen in East End and West Pond, can delay wetland greening by shading new growth. Differences in vegetation cover and canopy structure because of restoration design led to site‐specific variations in environmental factors driving fluxes. Although the diurnal and seasonal patterns of CO_2_ and CH_4_ fluxes are largely driven by environmental factors, site‐specific differences in conditions influenced the overall carbon balance of these wetlands. Wetland vegetation experiences regular seasonal cycles of growth and senescence, while the diurnal dynamics of physiological processes regulate the overall carbon uptake and emission by plants (Villa et al. 2020). Our results showed a strong correlation between the length of the carbon uptake period and the amount of CO_2_ sequestered, which is a result of delayed greening due to densely vegetated wetlands like West Pond, which was also progressively observed at East End due to the accumulation of dead biomass. This legacy vegetation insulates the understory affecting the magnitude and variability of water and soil temperatures (Eichelmann et al. 2018), thereby influencing vegetation growth and delaying the onset of the growing season (Dronova et al. 2021; Schile et al. 2013). For example, in the East End wetland, soil temperature became an increasingly important factor controlling CO_2_ uptake over time, as shown by MI and Theil‐Sen trend analyses. While soil temperature initially played a minor role due to sparse vegetation and smaller differences in air, water, and soil temperatures, the development of a dense canopy and biomass buildup eventually delayed spring soil warming to a degree like that observed at West Pond. Study by Sturtevant et al. (2016) also showed that on seasonal scales the soil and air temperature were among the most important variables for CH_4_ emissions.

Successful wetland restoration depends not only on hydrological re‐establishment but also on the timely recovery of wetland vegetation, which plays a crucial role in GHG regulation. Wetlands that failed to support rapid vegetation establishment due to either insufficient planting or unfavorable hydrological conditions remained substantial sources of both CO_2_ and CH_4_ years after restoration. For instance, Sherman Wetland in its first 3 years and Hill Slough Wetland have remained net GHG sources, primarily due to a lack of dense vegetative cover. Sherman Wetland exemplifies the challenges of unfavorable hydrological conditions. Inadequate inflow combined with high evaporation rates led to large areas of open water drying out during summer. This prevented the establishment of aquatic macrophytes and allowed terrestrial grasses to colonize these areas. However, these grasses were later inundated during autumn flooding and subsequently died, further disrupting ecosystem development and contributing to GHG emissions. Hill Slough, on the other hand, experiences too high water level during high tide and therefore suppresses the vegetation development as all the vegetation batches are located on higher ground, which experiences lower water level during high tide.

These two cases highlight how the absence of vegetation due to poor soil and hydrological conditions can delay restoration success, as seen in both sites. For example, even 3 years after restoration, Hill Slough remains a net GHG source, and model simulations indicate that it is unlikely to become a net GHG sink for several hundred years if the vegetation development is not speeding up. However, extensive planting may not always be necessary, particularly at sites where soils already contain high levels of natural seed banks as well as nutrients and carbon. For instance, initial soil carbon, nitrogen, and nutrient levels were significantly higher at East End compared to Sherman Wetland (Kasak et al. 2021), and vegetation establishment at East End was also more effective. The critical role of nutrients in wetland vegetation development and diversity was similarly demonstrated by Hong et al. (2021), who noted that nutrient availability was more important than site microtopography for revegetation. The Gilbert Tract wetland offers a compelling contrast to these outcomes. Thanks to deliberate soil modification and planting, this site became one of the largest CO_2_ sinks not only in the Delta but also on a global scale (Baldocchi et al. 2018) just 1 year after restoration. Although CH_4_ emissions also increased, the carbon uptake clearly dominates. However, the site's GHG dynamics are strongly influenced by the presence of *Azolla*, a rapidly growing, fast‐decomposing aquatic plant (Bastviken et al. 2023; Korsa et al. 2024) that forms dense mats over open water. *Azolla* contributes fresh carbon to the system (Ito and Watanabe 1983) while also limiting oxygen diffusion, making these areas important CH_4_ hotspots.

Soil carbon and nitrogen concentrations varied widely across sites, reflecting differences in wetland age, vegetation development, and site heterogeneity. A clear trend emerged: the older the wetland, the higher the carbon and nitrogen concentrations, placing these sites toward the upper end of global soil values reported by Batjes (2014). Mature wetlands (West Pond, Mayberry, and East End) had substantially higher carbon and nitrogen content than recently restored sites (Sherman Wetland, Gilbert Tract, and Hill Slough), consistent with long‐term organic matter accumulation under stable hydrological and vegetative conditions. These findings show that soil biogeochemical recovery is a gradual and long‐term process. Similar results have also been shown by (Moreno‐Mateos et al. 2012; Yu et al. 2017), who reported that decades to even a century after restoration, the soil carbon and nitrogen in restored wetlands was still significantly lower than in natural wetlands. Additionally, the tidal influence at Gilbert Tract and Hill Slough may contribute to lateral carbon losses (Arias‐Ortiz et al. 2021). We also noted that total annual CH_4_ emissions were negatively correlated with soil carbon and nitrogen content, suggesting that CH_4_ production is driven not only by carbon and nitrogen pools but also by other conditions associated with lower C/N systems, such as greater labile carbon availability, redox dynamics, and microbial competition (Bridgham et al. 2013).

### 4.3 Influence of Wetland Restoration on Long‐Term Climate Mitigation Goals

Delta restored wetlands have been modeled by several previous studies during recent years to predict switchover times, i.e., when the site becomes a net GHG sink, in comparison to previous land use types (Arias‐Ortiz et al. 2021; Hemes et al. 2019; Valach, Kasak, Hemes, Szutu, et al. 2021), and in each study, the prediction year changed due to the additional years of monitoring. Notably, the time required to transition from warming to cooling effects (crossing the zero line) appears to decrease for most sites as the monitoring duration increases from 3 to 10 years. This suggests that longer monitoring periods enable more accurate characterization of the interannual variability in biogeochemical processes driving GHG fluxes, resulting in more robust and, in this case, more optimistic projections of climate benefits. These results demonstrate that wetlands, which achieve maximum and high vegetation cover, and less year‐to‐year variability, likely have more accurate future predictions, as for example, East End projections did not differ significantly based on 3‐, 5‐, or 10‐year long measurement periods. In contrast, the prediction at Mayberry, which has a varying bathymetry and patchy vegetation cover, as well as experienced multiple external disturbances, showed that shorter measurement durations, i.e., 3 years, resulted in very long switchover estimations of 100 and 178 years compared with significantly shorter estimates of 43 and 71 years, after 10 years of measurements, based on instantaneous and cumulative radiative forcing, respectively. This was due to the flux variation in Mayberry over the years being the highest, which could still involve significant uncertainty in terms of actual switchover times, even after 10 years of measurements. For example, Mayberry CH_4_ emissions showed a clear declining trend for several years until 2024, when emissions abruptly returned to levels last observed 5 years earlier. This interruption in the trend coincides with findings from Delwiche et al. (2025), who reported a substantial drop in porewater conductivity in 2024, which returned to values similar in 2020 by a corresponding increase in CH_4_ emissions. This shows that even slight changes in some environmental parameters can drive resurgence in CH_4_ emissions. While the switchover time is predominantly affected by increased CH_4_ emissions, all future predictions for mature restored wetlands indicate that they should become net GHG sinks on climate‐relevant timescales. The study by Günther et al. (2020) similarly showed that CH_4_ radiative forcing does not undermine the long‐term mitigation potential of wetland restoration. However, in the short term, particularly over the next few decades that are critical for limiting global warming, restored wetlands can exhibit elevated CH_4_ emissions that may temporarily offset the CO_2_ benefits. In addition to CH_4_ dynamics, N_2_O also contributes to the overall radiative balance, though its role is less constrained in these systems. In estimating switchover time, we assume that N_2_O emissions decline sharply following restoration. This assumption is supported by short‐term campaigns indicating that restored Delta wetlands tend to act as small N_2_O sinks rather than sources (Kasak et al. 2021; McNicol et al. 2017), in contrast to agricultural lands, which are known to emit a significant amount of N_2_O (Anthony and Silver 2021; Anthony and Silvers 2024; Anthony et al. 2023).

Our study demonstrates that both the wetland restoration strategy and the construction approach pertaining to vegetation cover are critical for predicting successful outcomes. While rewetting drained soils can significantly reduce CO_2_ losses, it often leads to increased CH_4_ emissions. If vegetation fails to establish quickly, the site may continue to emit CO_2_, potentially becoming an even greater GHG source than the previous land use type until vegetation spreads. We also definitively show that although the post‐restoration increase in CH_4_ emissions reduces the short‐term climate mitigation potential, conditions with rapid vegetation growth, increased and sustained CO_2_ uptake can transform these sites into net GHG sinks in relatively short, climate relevant timescales. On the other hand, sites with tidal impact have slightly more complicated carbon accumulation rates due to the lateral transport (Arias‐Ortiz et al. 2021). In addition, effective site‐level management is essential, as disturbance events can severely impact CO_2_ uptake and delay the anticipated climate benefits as was clearly seen due to water level drawdown years in East End and West Pond. Therefore, sudden and persistent water level drawdowns, which expose carbon rich soils to aerobic conditions, will lead to the release of accumulated CO_2_. Encouragingly, our observations suggest that wetlands often recover quickly, even after substantial disturbances. Finally, we propose that when restored wetlands develop expansive, dense, and tall vegetation canopies, the timing of the transition to a net GHG sink can be predicted with high confidence. In contrast, sites with more heterogeneous vegetation patterns are harder to assess and forecast.

## Author Contributions

**Kuno Kasak:** conceptualization, data curation, formal analysis, funding acquisition, investigation, methodology, project administration, resources, software, validation, visualization, writing – original draft, writing – review and editing. **Arman Ahmadi:** data curation, formal analysis, methodology, writing – review and editing. **Iryna Dronova:** formal analysis, methodology, writing – review and editing. **Ariane Arias‐Ortiz:** formal analysis, writing – review and editing. **Tianxin Wang:** data curation, writing – review and editing. **Alex C. Valach:** writing – review and editing. **Daphne Szutu:** data curation. **Joseph Verfaillie:** data curation. **Dennis D. Baldocchi:** conceptualization, data curation, funding acquisition, project administration, resources, writing – review and editing.

## Conflicts of Interest

The authors declare no conflicts of interest.

## Data Availability

All sites used in this analysis are part of the Ameriflux network, with EC data available at http://ameriflux.lbl.gov/ using following DOIs: East End (https://doi.org/10.17190/AMF/2204881); West Pond (https://doi.org/10.17190/AMF/1832165); Sherman Wetland (https://doi.org/10.17190/AMF/1418684), Gilbert Tract (https://doi.org/10.17190/AMF/2469451); Hill Slough (https://doi.org/10.17190/AMF/2571131); and Mayberry (https://doi.org/10.17190/AMF/1871139).
